# *catena*-Poly[[bis­(μ_3_-5-hy­droxy­isophthalato)bis­(pyrazino­[2,3-*f*][1,10]phenanthroline)dicadmium] dihydrate]

**DOI:** 10.1107/S1600536812011695

**Published:** 2012-03-24

**Authors:** Peng-Cheng Zhao

**Affiliations:** a Department of Forensic Chemistry, China Criminal Police University, Shenyang 110854, People’s Republic of China

## Abstract

The title coordination polymer, {[Cd_2_(C_8_H_4_O_5_)_2_(C_14_H_8_N_4_)_2_]·2H_2_O}_*n*_, has a layered structure. The asymmetric unit contains two Cd^II^ ions, two pyrazino­[2,3-*f*][1,10]phenanthroline, two 5-hy­droxy­isophthalate (hip) ligands and two lattice water mol­ecules. Each Cd^II^ ion is coordinated by two N atoms from a chelating pyrazino­[2,3-*f*][1,10]phenanthroline and four O atoms from three different hip ligands, resulting in a distorted CdN_2_O_4_ octa­hedral coordination environment. The hip ligand connects adjacent Cd^II^ ions, forming forming layers parallel to (010). Intralayer O—H⋯O hydrogen bonds involving the hydroxy groups and solvent water molecules consolidate the crystal packing.

## Related literature

For metal–carboxyl­ate complexes containing a pyrazino­[2,3-*f*][1,10]phenanthroline ligand, see: He & Han (2006 ▶); Han *et al.* (2009 ▶); Wang *et al.* (2007 ▶). 
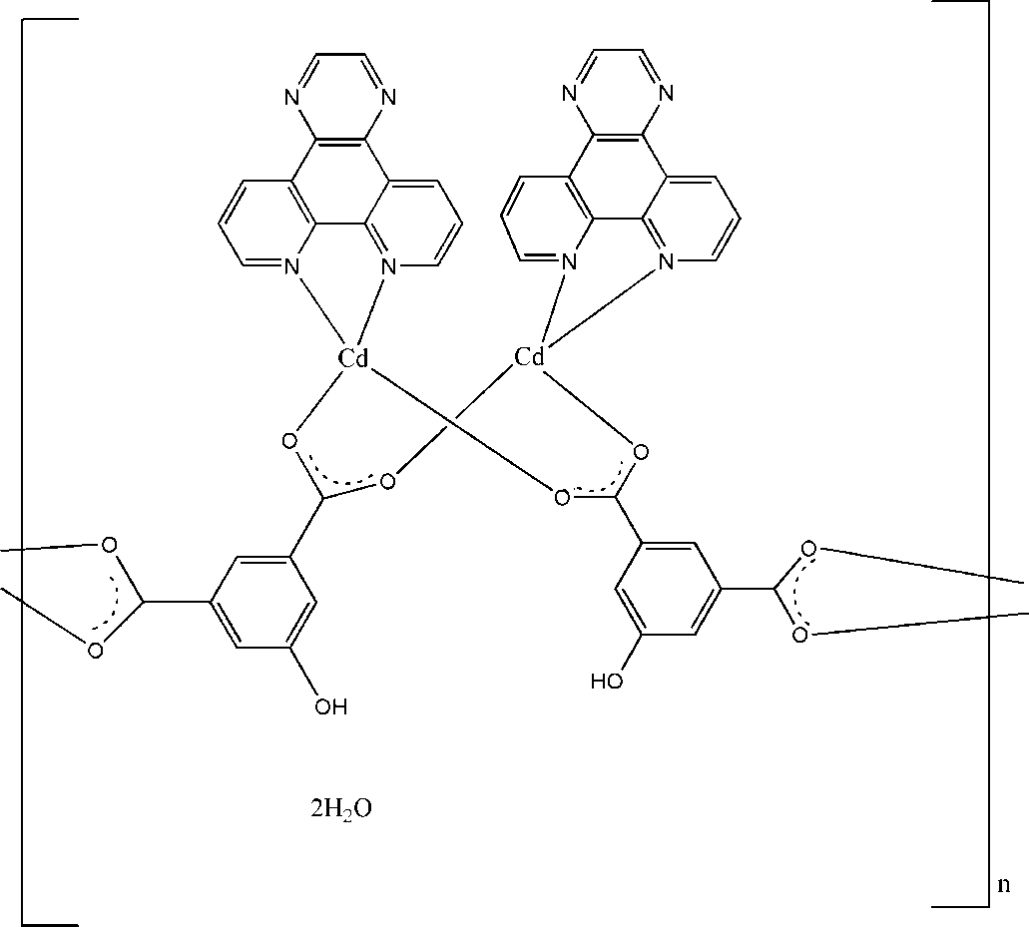


## Experimental

### 

#### Crystal data

[Cd_2_(C_8_H_4_O_5_)_2_(C_14_H_8_N_4_)_2_]·2H_2_O
*M* *_r_* = 1085.54
Triclinic, 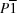

*a* = 8.6754 (13) Å
*b* = 15.1114 (17) Å
*c* = 15.629 (3) Å
α = 92.903 (16)°
β = 97.143 (13)°
γ = 95.515 (9)°
*V* = 2019.6 (5) Å^3^
*Z* = 2
Mo *K*α radiation
μ = 1.13 mm^−1^
*T* = 293 K
0.37 × 0.33 × 0.27 mm

#### Data collection

Bruker APEX area-detector diffractometer
Absorption correction: multi-scan (*SADABS*; Sheldrick, 1996 ▶) *T*_min_ = 0.681, *T*_max_ = 0.751
11100 measured reflections
9275 independent reflections
8147 reflections with *I* > 2σ(*I*)
*R*_int_ = 0.061

#### Refinement

*R*[*F* ^2^ > 2σ(*F* ^2^)] = 0.031
*wR*(*F* ^2^) = 0.114
*S* = 1.07
9275 reflections
595 parameters
H-atom parameters constrained
Δρ_max_ = 0.70 e Å^−3^
Δρ_min_ = −1.43 e Å^−3^

### 

Data collection: *SMART* (Bruker, 2001 ▶); cell refinement: *SAINT* (Bruker, 2001 ▶); data reduction: *SAINT*; program(s) used to solve structure: *SHELXS97* (Sheldrick, 2008 ▶); program(s) used to refine structure: *SHELXL97* (Sheldrick, 2008 ▶); molecular graphics: *SHELXTL* (Sheldrick, 2008 ▶); software used to prepare material for publication: *SHELXL97*.

## Supplementary Material

Crystal structure: contains datablock(s) I, global. DOI: 10.1107/S1600536812011695/ds2166sup1.cif

Structure factors: contains datablock(s) I. DOI: 10.1107/S1600536812011695/ds2166Isup2.hkl

Additional supplementary materials:  crystallographic information; 3D view; checkCIF report

## Figures and Tables

**Table 1:** Selected bond lengths (Å)

Cd1—O7	2.205 (3)
Cd1—O1	2.259 (2)
Cd1—N2	2.339 (3)
Cd1—O4^i^	2.360 (2)
Cd1—N1	2.367 (3)
Cd1—O5^i^	2.384 (3)
Cd2—O2	2.202 (2)
Cd2—O6	2.295 (3)
Cd2—N5	2.325 (3)
Cd2—O9^ii^	2.332 (2)
Cd2—N6	2.343 (3)
Cd2—O10^ii^	2.362 (3)

Symmetry codes: (i) 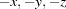; (ii) 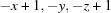.

**Table 2:** Hydrogen-bond geometry (Å, °)

*D*—H⋯*A*	*D*—H	H⋯*A*	*D*⋯*A*	*D*—H⋯*A*
O3—H3*B*⋯O2*W*^iii^	0.82	1.84	2.659 (4)	177
O8—H8*A*⋯O1*W*^iv^	0.82	1.85	2.672 (4)	177
O1*W*—H1*WA*⋯O9^v^	0.85	2.14	2.912 (4)	150
O1*W*—H1*WB*⋯O2^v^	0.85	2.27	2.963 (4)	138
O2*W*—H2*WA*⋯O4^vi^	0.85	2.28	2.880 (4)	127
O2*W*—H2*WB*⋯O7^vi^	0.85	2.33	2.955 (4)	131

Symmetry codes: (iii) 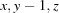; (iv) 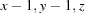; (v) 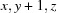; (vi) 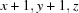.

**Fig. 1.**
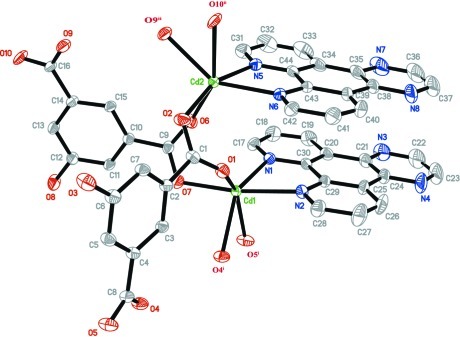
View of the structure of (I). Displacement ellipsoids are drawn at the 30% probability level. H atoms and the lattice water molecule have been omitted for clarity. [Symmetry code: (i) -x, -y + 1, -z + 1.]

**Fig. 2.**
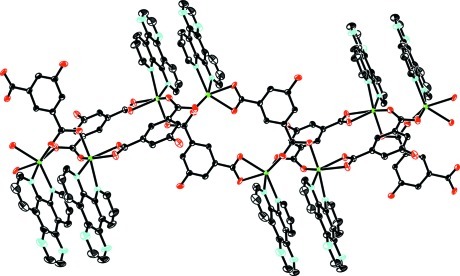
The fragment of one-dimensional ribbon chain.

## supplementary crystallographic information

### Comment

Recently, several metal-carboxylate complexes containing an N-donor chelate ligand TATP and its large analogue DPPZ dipyridophenazine have been reported (He & Han, 2006; Wang *et al.*, 2007; Han *et al.*, 2009). I report here a new one-dimensional Cd^II^ coordination polymer constructed by Cd^II^ ions, pyrazino[2,3-*f*][1,10]phenanthroline (TATP) and 5-hydroxyisophthalic acid (H_2_Hip), (I).

Complex (I) exhibits a layered structure in which the asymmetric unit consists of two Cd^II^ ions, two hip, two TATP ligand and two lattice water molecules (Fig. 1). Fig. 2 shows a fragment of the fragment of ribbon chain in the structure of I. Each Cd^II^ is hexa-coordinate and is surrounded by four oxygen atoms from three different hip ligands and two nitrogen atoms from a chelating TATP ligand (Table 1), forming a distorted octahedral geometry.

The hip ligand connects adjacent Cd^II^ ions, forming layers parallel to (010). Intralayer O—H···O hydrogen bonds involving the hydroxy groups and solvent water molecules consolidate the crystal packing. A face-to-face distance of 3.491 Å between a pair of TATP ligands coordinated to the two Cd^II^ ions is observed, showing significant π–π stacking interactions.

### Experimental

A mixture of Cd(NO_3_)_2_.4H_2_O (0.5 mmol, 0.154 g), pyrazino[2,3-*f*][1,10]phenanthroline ligand (0.5 mmol, 0.116 g), H_2_hip (0.5 mmol, 0.083 g) and water (10 ml) was mixed in a 23 ml Teflon reactor, which was heated at 180° for six days and then cooled to room temperature at a rate of 5 ° h^-1^.Yield: 38%. CH&N analysis for C_68_H_38_Cu_3_N_8_ (found/calc): C, 48.68(48.97), H, 2.60(2.71), N, 10.32%(10.63%).

### Refinement

The H atoms of the aromatic rings were placed at calculated positions in the riding model approximation (C—H 0.93 Å) with their temperature factors were set to 1.2 times those of the equivalent isotropic temperature factors of the parent atoms. The hydroxy H atom was placed at calculated positions in the riding model approximation(O—H 0.82 Å) with their temperature factors were set to 1.2 times those of the equivalent isotropic temperature factors of the parent atoms.

### Crystal data

**Table:**

[Cd_2_(C_8_H_4_O_5_)_2_(C_14_H_8_N_4_)_2_]·2H_2_O	*Z* = 2
*M**_r_* = 1085.54	*F*(000) = 1080
Triclinic, *P*1	*D*_x_ = 1.785 Mg m^−^^3^
Hall symbol: -P 1	Mo *K*α radiation, λ = 0.71073 Å
*a* = 8.6754 (13) Å	Cell parameters from 20 reflections
*b* = 15.1114 (17) Å	θ = 2.7–22.3°
*c* = 15.629 (3) Å	µ = 1.13 mm^−^^1^
α = 92.903 (16)°	*T* = 293 K
β = 97.143 (13)°	Block, red
γ = 95.515 (9)°	0.37 × 0.33 × 0.27 mm
*V* = 2019.6 (5) Å^3^	

### Data collection

**Table:**

Bruker APEX area-detector diffractometer	9275 independent reflections
Radiation source: fine-focus sealed tube	8147 reflections with *I* > 2σ(*I*)
Graphite monochromator	*R*_int_ = 0.061
φ and ω scan	θ_max_ = 27.5°, θ_min_ = 1.8°
Absorption correction: multi-scan (*SADABS*; Sheldrick, 1996)	*h* = −1→11
*T*_min_ = 0.681, *T*_max_ = 0.751	*k* = −19→19
11100 measured reflections	*l* = −20→20

### Refinement

**Table:**

Refinement on *F*^2^	Primary atom site location: structure-invariant direct methods
Least-squares matrix: full	Secondary atom site location: difference Fourier map
*R*[*F*^2^ > 2σ(*F*^2^)] = 0.031	Hydrogen site location: inferred from neighbouring sites
*wR*(*F*^2^) = 0.114	H-atom parameters constrained
*S* = 1.07	*w* = 1/[σ^2^(*F*_o_^2^) + (0.0613*P*)^2^ + 2.6404*P*] where *P* = (*F*_o_^2^ + 2*F*_c_^2^)/3
9275 reflections	(Δ/σ)_max_ = 0.009
595 parameters	Δρ_max_ = 0.70 e Å^−^^3^
0 restraints	Δρ_min_ = −1.43 e Å^−^^3^

### Special details

**Table:**

Geometry. All e.s.d.'s (except the e.s.d. in the dihedral angle between two l.s. planes) are estimated using the full covariance matrix. The cell e.s.d.'s are taken into account individually in the estimation of e.s.d.'s in distances, angles and torsion angles; correlations between e.s.d.'s in cell parameters are only used when they are defined by crystal symmetry. An approximate (isotropic) treatment of cell e.s.d.'s is used for estimating e.s.d.'s involving l.s. planes.
Refinement. Refinement of *F*^2^ against ALL reflections. The weighted *R*-factor *wR* and goodness of fit *S* are based on *F*^2^, conventional *R*-factors *R* are based on *F*, with *F* set to zero for negative *F*^2^. The threshold expression of *F*^2^ > σ(*F*^2^) is used only for calculating *R*-factors(gt) *etc*. and is not relevant to the choice of reflections for refinement. *R*-factors based on *F*^2^ are statistically about twice as large as those based on *F*, and *R*- factors based on ALL data will be even larger.

### Fractional atomic coordinates and isotropic or equivalent isotropic displacement parameters (Å^2^)

**Table:**

	*x*	*y*	*z*	*U*_iso_*/*U*_eq_	
Cd1	0.10447 (3)	0.188967 (14)	0.170325 (14)	0.02701 (8)	
Cd2	0.47056 (3)	0.185064 (14)	0.355404 (14)	0.02830 (8)	
C1	0.4157 (4)	0.0769 (2)	0.1864 (2)	0.0325 (7)	
C2	0.4157 (4)	−0.0043 (2)	0.1274 (2)	0.0293 (6)	
C3	0.2868 (4)	−0.0312 (2)	0.0662 (2)	0.0298 (6)	
H3A	0.2047	0.0041	0.0578	0.036*	
C4	0.2821 (4)	−0.1116 (2)	0.0176 (2)	0.0306 (6)	
C5	0.4080 (4)	−0.1622 (2)	0.0279 (2)	0.0355 (7)	
H5A	0.4056	−0.2151	−0.0055	0.043*	
C6	0.5372 (4)	−0.1341 (2)	0.0879 (2)	0.0359 (7)	
C7	0.5410 (4)	−0.0553 (2)	0.1379 (2)	0.0327 (7)	
H7A	0.6272	−0.0366	0.1785	0.039*	
C8	0.1421 (4)	−0.1441 (2)	−0.0463 (2)	0.0328 (7)	
C9	0.1081 (4)	0.0759 (2)	0.3173 (2)	0.0327 (7)	
C10	0.0805 (4)	−0.0039 (2)	0.3672 (2)	0.0301 (6)	
C11	−0.0632 (4)	−0.0553 (2)	0.3504 (2)	0.0335 (7)	
H11A	−0.1422	−0.0371	0.3114	0.040*	
C12	−0.0887 (4)	−0.1337 (2)	0.3918 (2)	0.0355 (7)	
C13	0.0304 (4)	−0.1612 (2)	0.4484 (2)	0.0362 (7)	
H13A	0.0143	−0.2141	0.4755	0.043*	
C14	0.1743 (4)	−0.1098 (2)	0.4649 (2)	0.0306 (6)	
C15	0.1995 (4)	−0.0305 (2)	0.4259 (2)	0.0297 (6)	
H15A	0.2944	0.0047	0.4386	0.036*	
C16	0.3017 (4)	−0.1412 (2)	0.5267 (2)	0.0314 (6)	
C17	−0.0218 (5)	0.3044 (3)	0.3270 (2)	0.0407 (8)	
H17A	−0.0325	0.2492	0.3506	0.049*	
C18	−0.0821 (6)	0.3758 (3)	0.3664 (3)	0.0544 (11)	
H18A	−0.1310	0.3686	0.4156	0.065*	
C19	−0.0680 (6)	0.4571 (3)	0.3312 (3)	0.0565 (11)	
H19A	−0.1064	0.5058	0.3568	0.068*	
C20	0.0046 (5)	0.4664 (2)	0.2565 (3)	0.0419 (8)	
C21	0.0198 (5)	0.5501 (3)	0.2146 (3)	0.0509 (10)	
C22	−0.0273 (9)	0.6946 (3)	0.2073 (5)	0.090 (2)	
H22A	−0.0683	0.7450	0.2279	0.108*	
C23	0.0499 (9)	0.6999 (3)	0.1355 (6)	0.097 (2)	
H23A	0.0581	0.7540	0.1098	0.117*	
C24	0.0961 (6)	0.5557 (3)	0.1413 (3)	0.0531 (11)	
C25	0.1584 (5)	0.4778 (3)	0.1055 (3)	0.0439 (9)	
C26	0.2386 (7)	0.4795 (4)	0.0330 (3)	0.0671 (14)	
H26A	0.2538	0.5320	0.0052	0.081*	
C27	0.2944 (7)	0.4036 (4)	0.0034 (4)	0.0712 (15)	
H27A	0.3496	0.4044	−0.0440	0.085*	
C28	0.2678 (5)	0.3250 (3)	0.0449 (3)	0.0495 (10)	
H28A	0.3031	0.2731	0.0235	0.059*	
C29	0.1410 (4)	0.3973 (2)	0.1447 (2)	0.0329 (7)	
C30	0.0625 (4)	0.3913 (2)	0.2218 (2)	0.0317 (6)	
C31	0.3204 (5)	0.3164 (3)	0.4855 (3)	0.0500 (10)	
H31A	0.3056	0.2645	0.5143	0.060*	
C32	0.2675 (6)	0.3944 (4)	0.5176 (4)	0.0709 (15)	
H32A	0.2197	0.3945	0.5677	0.085*	
C33	0.2864 (6)	0.4703 (4)	0.4750 (4)	0.0718 (16)	
H33A	0.2503	0.5223	0.4954	0.086*	
C34	0.3604 (5)	0.4699 (3)	0.4003 (3)	0.0499 (10)	
C35	0.3880 (6)	0.5474 (3)	0.3515 (4)	0.0602 (14)	
C36	0.3659 (10)	0.6943 (4)	0.3319 (7)	0.105 (3)	
H36A	0.3323	0.7486	0.3482	0.126*	
C37	0.4468 (10)	0.6902 (4)	0.2608 (6)	0.104 (3)	
H37A	0.4666	0.7415	0.2318	0.125*	
C38	0.4669 (6)	0.5434 (3)	0.2791 (3)	0.0574 (12)	
C39	0.5212 (5)	0.4600 (2)	0.2509 (3)	0.0457 (9)	
C40	0.6034 (6)	0.4512 (3)	0.1801 (3)	0.0616 (13)	
H40A	0.6253	0.5000	0.1481	0.074*	
C41	0.6517 (6)	0.3701 (3)	0.1580 (3)	0.0574 (11)	
H41A	0.7090	0.3641	0.1119	0.069*	
C42	0.6148 (5)	0.2978 (3)	0.2045 (2)	0.0419 (8)	
H42A	0.6449	0.2428	0.1879	0.050*	
C43	0.4924 (4)	0.3842 (2)	0.2967 (2)	0.0331 (7)	
C44	0.4124 (4)	0.3889 (2)	0.3731 (2)	0.0331 (7)	
N1	0.0498 (3)	0.31172 (18)	0.25765 (18)	0.0313 (6)	
N2	0.1936 (3)	0.32205 (19)	0.11392 (19)	0.0347 (6)	
N3	−0.0450 (6)	0.6199 (3)	0.2481 (3)	0.0731 (13)	
N4	0.1131 (6)	0.6324 (3)	0.1010 (4)	0.0768 (14)	
N5	0.3913 (3)	0.3145 (2)	0.41515 (18)	0.0341 (6)	
N6	0.5371 (3)	0.30456 (19)	0.27266 (18)	0.0320 (6)	
N7	0.3338 (6)	0.6247 (3)	0.3780 (4)	0.0876 (17)	
N8	0.4967 (6)	0.6145 (3)	0.2330 (4)	0.0839 (16)	
O1	0.3399 (3)	0.13916 (16)	0.16202 (18)	0.0387 (5)	
O2	0.4944 (3)	0.07607 (18)	0.26051 (17)	0.0445 (6)	
O3	0.6581 (3)	−0.18602 (19)	0.0963 (2)	0.0507 (7)	
H3B	0.7409	−0.1541	0.1070	0.076*	
O4	0.0151 (3)	−0.11105 (18)	−0.04066 (16)	0.0396 (6)	
O5	0.1536 (3)	−0.2029 (2)	−0.10328 (18)	0.0463 (7)	
O6	0.2090 (3)	0.13873 (17)	0.34703 (19)	0.0411 (6)	
O7	0.0264 (3)	0.07608 (18)	0.24391 (18)	0.0452 (6)	
O8	−0.2273 (3)	−0.1863 (2)	0.3755 (2)	0.0531 (8)	
H8A	−0.2992	−0.1548	0.3689	0.080*	
O9	0.4413 (3)	−0.10890 (18)	0.52495 (17)	0.0418 (6)	
O10	0.2685 (3)	−0.1985 (2)	0.57699 (19)	0.0488 (7)	
O1W	0.5320 (3)	0.9117 (2)	0.3532 (2)	0.0517 (7)	
H1WA	0.4828	0.9197	0.3963	0.062*	
H1WB	0.5729	0.9562	0.3299	0.062*	
O2W	0.9326 (3)	0.91177 (19)	0.1314 (2)	0.0482 (6)	
H2WA	0.9958	0.8862	0.1036	0.058*	
H2WB	0.9335	0.9681	0.1328	0.058*	

### Atomic displacement parameters (Å^2^)

**Table:**

	*U*^11^	*U*^22^	*U*^33^	*U*^12^	*U*^13^	*U*^23^
Cd1	0.02968 (12)	0.02234 (12)	0.02818 (12)	0.00372 (8)	−0.00040 (9)	0.00180 (8)
Cd2	0.03162 (13)	0.02485 (12)	0.02733 (12)	0.00397 (9)	−0.00178 (9)	0.00298 (8)
C1	0.0262 (15)	0.0321 (16)	0.0382 (17)	0.0037 (12)	0.0032 (13)	−0.0048 (13)
C2	0.0280 (15)	0.0315 (15)	0.0284 (15)	0.0067 (12)	0.0022 (12)	−0.0014 (12)
C3	0.0294 (15)	0.0305 (15)	0.0298 (15)	0.0079 (12)	0.0011 (12)	0.0012 (12)
C4	0.0290 (15)	0.0341 (16)	0.0281 (15)	0.0040 (12)	0.0019 (12)	−0.0021 (12)
C5	0.0362 (17)	0.0334 (16)	0.0359 (17)	0.0079 (13)	0.0015 (14)	−0.0088 (13)
C6	0.0283 (15)	0.0396 (18)	0.0401 (18)	0.0119 (13)	0.0013 (13)	−0.0032 (14)
C7	0.0272 (15)	0.0364 (17)	0.0330 (16)	0.0072 (13)	−0.0024 (12)	−0.0048 (13)
C8	0.0303 (16)	0.0371 (17)	0.0293 (15)	0.0026 (13)	−0.0010 (12)	−0.0014 (13)
C9	0.0285 (15)	0.0310 (16)	0.0390 (17)	0.0039 (12)	0.0038 (13)	0.0077 (13)
C10	0.0265 (14)	0.0309 (15)	0.0329 (16)	0.0029 (12)	0.0024 (12)	0.0053 (12)
C11	0.0255 (15)	0.0358 (17)	0.0382 (17)	0.0015 (12)	−0.0028 (13)	0.0112 (14)
C12	0.0271 (15)	0.0357 (17)	0.0428 (18)	−0.0020 (13)	0.0026 (13)	0.0091 (14)
C13	0.0330 (16)	0.0370 (17)	0.0390 (18)	0.0014 (13)	0.0028 (14)	0.0143 (14)
C14	0.0287 (15)	0.0362 (16)	0.0269 (14)	0.0041 (12)	0.0016 (12)	0.0080 (12)
C15	0.0251 (14)	0.0329 (16)	0.0302 (15)	0.0013 (12)	−0.0010 (12)	0.0066 (12)
C16	0.0317 (16)	0.0353 (16)	0.0269 (15)	0.0059 (13)	−0.0019 (12)	0.0074 (12)
C17	0.049 (2)	0.045 (2)	0.0304 (17)	0.0088 (16)	0.0089 (15)	0.0065 (14)
C18	0.066 (3)	0.059 (3)	0.042 (2)	0.013 (2)	0.022 (2)	−0.0035 (19)
C19	0.066 (3)	0.052 (2)	0.053 (2)	0.019 (2)	0.013 (2)	−0.014 (2)
C20	0.0424 (19)	0.0328 (17)	0.048 (2)	0.0073 (15)	−0.0021 (16)	−0.0064 (15)
C21	0.055 (2)	0.0277 (17)	0.067 (3)	0.0118 (16)	−0.009 (2)	−0.0003 (17)
C22	0.111 (5)	0.032 (2)	0.122 (6)	0.030 (3)	−0.015 (4)	−0.004 (3)
C23	0.116 (6)	0.030 (2)	0.141 (7)	0.017 (3)	−0.015 (5)	0.023 (3)
C24	0.059 (3)	0.0320 (19)	0.065 (3)	0.0057 (17)	−0.010 (2)	0.0135 (18)
C25	0.047 (2)	0.0348 (18)	0.049 (2)	0.0035 (15)	−0.0007 (17)	0.0165 (16)
C26	0.082 (4)	0.060 (3)	0.064 (3)	0.004 (3)	0.019 (3)	0.038 (2)
C27	0.087 (4)	0.072 (3)	0.066 (3)	0.013 (3)	0.041 (3)	0.028 (3)
C28	0.057 (2)	0.052 (2)	0.044 (2)	0.0081 (19)	0.0199 (19)	0.0089 (18)
C29	0.0320 (16)	0.0285 (15)	0.0371 (17)	0.0030 (12)	−0.0003 (13)	0.0034 (13)
C30	0.0304 (15)	0.0273 (15)	0.0362 (16)	0.0024 (12)	0.0007 (13)	0.0001 (12)
C31	0.049 (2)	0.061 (3)	0.040 (2)	0.0011 (19)	0.0132 (17)	−0.0023 (18)
C32	0.061 (3)	0.084 (4)	0.069 (3)	0.006 (3)	0.029 (3)	−0.027 (3)
C33	0.059 (3)	0.065 (3)	0.091 (4)	0.016 (2)	0.015 (3)	−0.035 (3)
C34	0.040 (2)	0.038 (2)	0.067 (3)	0.0092 (16)	−0.0071 (18)	−0.0175 (18)
C35	0.052 (2)	0.0293 (19)	0.090 (4)	0.0079 (17)	−0.025 (2)	−0.010 (2)
C36	0.108 (6)	0.027 (2)	0.164 (8)	0.015 (3)	−0.047 (5)	−0.005 (4)
C37	0.122 (6)	0.029 (2)	0.147 (7)	0.003 (3)	−0.037 (5)	0.018 (3)
C38	0.061 (3)	0.0285 (18)	0.075 (3)	0.0010 (17)	−0.023 (2)	0.0097 (19)
C39	0.050 (2)	0.0318 (18)	0.050 (2)	−0.0046 (16)	−0.0110 (17)	0.0113 (16)
C40	0.072 (3)	0.055 (3)	0.054 (3)	−0.014 (2)	0.002 (2)	0.025 (2)
C41	0.067 (3)	0.067 (3)	0.038 (2)	−0.008 (2)	0.016 (2)	0.009 (2)
C42	0.048 (2)	0.046 (2)	0.0312 (17)	0.0009 (16)	0.0083 (15)	0.0050 (15)
C43	0.0330 (16)	0.0294 (15)	0.0342 (16)	0.0000 (12)	−0.0047 (13)	0.0035 (13)
C44	0.0308 (16)	0.0288 (15)	0.0373 (17)	0.0026 (12)	−0.0027 (13)	−0.0042 (13)
N1	0.0336 (14)	0.0295 (13)	0.0307 (13)	0.0040 (11)	0.0041 (11)	0.0008 (11)
N2	0.0370 (15)	0.0308 (14)	0.0368 (15)	0.0023 (11)	0.0073 (12)	0.0021 (11)
N3	0.086 (3)	0.041 (2)	0.091 (3)	0.028 (2)	−0.006 (3)	−0.010 (2)
N4	0.092 (3)	0.039 (2)	0.101 (4)	0.010 (2)	0.002 (3)	0.032 (2)
N5	0.0356 (14)	0.0360 (15)	0.0300 (14)	0.0032 (12)	0.0029 (11)	0.0006 (11)
N6	0.0370 (15)	0.0312 (14)	0.0273 (13)	0.0026 (11)	0.0025 (11)	0.0014 (11)
N7	0.086 (3)	0.040 (2)	0.129 (5)	0.022 (2)	−0.019 (3)	−0.023 (3)
N8	0.097 (4)	0.034 (2)	0.110 (4)	−0.008 (2)	−0.027 (3)	0.025 (2)
O1	0.0341 (12)	0.0315 (12)	0.0504 (15)	0.0095 (10)	0.0017 (11)	−0.0017 (11)
O2	0.0493 (15)	0.0424 (14)	0.0387 (14)	0.0153 (12)	−0.0084 (11)	−0.0117 (11)
O3	0.0348 (13)	0.0447 (15)	0.0699 (19)	0.0169 (11)	−0.0047 (13)	−0.0188 (14)
O4	0.0324 (12)	0.0468 (14)	0.0371 (13)	0.0101 (10)	−0.0058 (10)	−0.0080 (11)
O5	0.0378 (14)	0.0566 (17)	0.0409 (14)	0.0113 (12)	−0.0061 (11)	−0.0168 (12)
O6	0.0342 (13)	0.0321 (12)	0.0549 (16)	−0.0020 (10)	0.0019 (11)	0.0042 (11)
O7	0.0449 (15)	0.0430 (14)	0.0442 (15)	−0.0060 (12)	−0.0050 (12)	0.0161 (12)
O8	0.0294 (13)	0.0476 (16)	0.080 (2)	−0.0081 (11)	−0.0032 (13)	0.0275 (15)
O9	0.0314 (12)	0.0500 (15)	0.0428 (14)	0.0010 (11)	−0.0044 (10)	0.0201 (12)
O10	0.0400 (14)	0.0602 (17)	0.0460 (15)	0.0017 (12)	−0.0053 (11)	0.0358 (13)
O1W	0.0513 (16)	0.0541 (17)	0.0541 (17)	0.0138 (13)	0.0142 (13)	0.0125 (14)
O2W	0.0439 (15)	0.0442 (15)	0.0571 (17)	0.0021 (12)	0.0106 (13)	0.0023 (13)

### Geometric parameters (Å, º)

**Table:**

Cd1—O7	2.205 (3)	C21—C24	1.395 (7)
Cd1—O1	2.259 (2)	C22—N3	1.331 (8)
Cd1—N2	2.339 (3)	C22—C23	1.377 (11)
Cd1—O4^i^	2.360 (2)	C22—H22A	0.9300
Cd1—N1	2.367 (3)	C23—N4	1.326 (9)
Cd1—O5^i^	2.384 (3)	C23—H23A	0.9300
Cd1—C8^i^	2.711 (3)	C24—N4	1.351 (6)
Cd2—O2	2.202 (2)	C24—C25	1.457 (6)
Cd2—O6	2.295 (3)	C25—C29	1.393 (5)
Cd2—N5	2.325 (3)	C25—C26	1.402 (7)
Cd2—O9^ii^	2.332 (2)	C26—C27	1.369 (8)
Cd2—N6	2.343 (3)	C26—H26A	0.9300
Cd2—O10^ii^	2.362 (3)	C27—C28	1.396 (6)
Cd2—C16^ii^	2.685 (3)	C27—H27A	0.9300
C1—O1	1.247 (4)	C28—N2	1.323 (5)
C1—O2	1.271 (4)	C28—H28A	0.9300
C1—C2	1.497 (4)	C29—N2	1.353 (4)
C2—C7	1.390 (4)	C29—C30	1.459 (5)
C2—C3	1.392 (4)	C30—N1	1.353 (4)
C3—C4	1.394 (4)	C31—N5	1.325 (5)
C3—H3A	0.9300	C31—C32	1.397 (7)
C4—C5	1.391 (5)	C31—H31A	0.9300
C4—C8	1.499 (4)	C32—C33	1.362 (9)
C5—C6	1.386 (5)	C32—H32A	0.9300
C5—H5A	0.9300	C33—C34	1.400 (8)
C6—O3	1.366 (4)	C33—H33A	0.9300
C6—C7	1.386 (5)	C34—C44	1.408 (5)
C7—H7A	0.9300	C34—C35	1.447 (7)
C8—O5	1.245 (4)	C35—N7	1.365 (6)
C8—O4	1.264 (4)	C35—C38	1.396 (8)
C8—Cd1^i^	2.711 (3)	C36—N7	1.332 (10)
C9—O6	1.257 (4)	C36—C37	1.389 (12)
C9—O7	1.272 (4)	C36—H36A	0.9300
C9—C10	1.485 (4)	C37—N8	1.333 (9)
C10—C11	1.392 (4)	C37—H37A	0.9300
C10—C15	1.397 (4)	C38—N8	1.346 (6)
C11—C12	1.392 (5)	C38—C39	1.454 (6)
C11—H11A	0.9300	C39—C40	1.396 (7)
C12—O8	1.365 (4)	C39—C43	1.400 (5)
C12—C13	1.383 (5)	C40—C41	1.375 (7)
C13—C14	1.394 (5)	C40—H40A	0.9300
C13—H13A	0.9300	C41—C42	1.376 (6)
C14—C15	1.383 (4)	C41—H41A	0.9300
C14—C16	1.503 (4)	C42—N6	1.335 (5)
C15—H15A	0.9300	C42—H42A	0.9300
C16—O10	1.237 (4)	C43—N6	1.348 (4)
C16—O9	1.266 (4)	C43—C44	1.455 (5)
C16—Cd2^ii^	2.685 (3)	C44—N5	1.339 (5)
C17—N1	1.320 (5)	O3—H3B	0.8200
C17—C18	1.392 (6)	O4—Cd1^i^	2.360 (2)
C17—H17A	0.9300	O5—Cd1^i^	2.384 (3)
C18—C19	1.372 (7)	O8—H8A	0.8200
C18—H18A	0.9300	O9—Cd2^ii^	2.332 (2)
C19—C20	1.400 (6)	O10—Cd2^ii^	2.362 (3)
C19—H19A	0.9300	O1W—H1WA	0.8500
C20—C30	1.396 (5)	O1W—H1WB	0.8500
C20—C21	1.457 (6)	O2W—H2WA	0.8500
C21—N3	1.354 (6)	O2W—H2WB	0.8499
			
O7—Cd1—O1	92.68 (10)	C20—C19—H19A	120.1
O7—Cd1—N2	170.68 (10)	C30—C20—C19	117.5 (4)
O1—Cd1—N2	89.39 (10)	C30—C20—C21	119.9 (4)
O7—Cd1—O4^i^	89.95 (10)	C19—C20—C21	122.5 (4)
O1—Cd1—O4^i^	94.29 (9)	N3—C21—C24	122.4 (4)
N2—Cd1—O4^i^	98.96 (10)	N3—C21—C20	117.7 (5)
O7—Cd1—N1	101.48 (11)	C24—C21—C20	119.9 (4)
O1—Cd1—N1	127.16 (9)	N3—C22—C23	122.1 (5)
N2—Cd1—N1	70.20 (10)	N3—C22—H22A	118.9
O4^i^—Cd1—N1	135.72 (9)	C23—C22—H22A	118.9
O7—Cd1—O5^i^	92.96 (11)	N4—C23—C22	123.9 (5)
O1—Cd1—O5^i^	148.77 (9)	N4—C23—H23A	118.0
N2—Cd1—O5^i^	89.96 (11)	C22—C23—H23A	118.0
O4^i^—Cd1—O5^i^	55.03 (9)	N4—C24—C21	121.5 (4)
N1—Cd1—O5^i^	81.52 (9)	N4—C24—C25	118.2 (5)
O7—Cd1—C8^i^	90.03 (10)	C21—C24—C25	120.3 (4)
O1—Cd1—C8^i^	122.03 (10)	C29—C25—C26	117.2 (4)
N2—Cd1—C8^i^	96.58 (11)	C29—C25—C24	119.7 (4)
O4^i^—Cd1—C8^i^	27.78 (9)	C26—C25—C24	123.0 (4)
N1—Cd1—C8^i^	108.73 (10)	C27—C26—C25	119.7 (4)
O5^i^—Cd1—C8^i^	27.34 (10)	C27—C26—H26A	120.1
O2—Cd2—O6	89.39 (10)	C25—C26—H26A	120.1
O2—Cd2—N5	160.40 (10)	C26—C27—C28	119.4 (4)
O6—Cd2—N5	81.94 (10)	C26—C27—H27A	120.3
O2—Cd2—O9^ii^	94.61 (10)	C28—C27—H27A	120.3
O6—Cd2—O9^ii^	98.31 (9)	N2—C28—C27	121.9 (4)
N5—Cd2—O9^ii^	103.99 (10)	N2—C28—H28A	119.1
O2—Cd2—N6	97.96 (11)	C27—C28—H28A	119.1
O6—Cd2—N6	116.05 (10)	N2—C29—C25	122.7 (3)
N5—Cd2—N6	70.66 (10)	N2—C29—C30	117.1 (3)
O9^ii^—Cd2—N6	143.34 (10)	C25—C29—C30	120.2 (3)
O2—Cd2—O10^ii^	96.45 (11)	N1—C30—C20	122.3 (3)
O6—Cd2—O10^ii^	153.41 (9)	N1—C30—C29	117.8 (3)
N5—Cd2—O10^ii^	99.14 (11)	C20—C30—C29	119.9 (3)
O9^ii^—Cd2—O10^ii^	55.45 (9)	N5—C31—C32	121.7 (5)
N6—Cd2—O10^ii^	88.88 (9)	N5—C31—H31A	119.1
O2—Cd2—C16^ii^	94.70 (10)	C32—C31—H31A	119.1
O6—Cd2—C16^ii^	126.42 (10)	C33—C32—C31	119.5 (5)
N5—Cd2—C16^ii^	104.61 (10)	C33—C32—H32A	120.3
O9^ii^—Cd2—C16^ii^	28.12 (9)	C31—C32—H32A	120.3
N6—Cd2—C16^ii^	116.16 (10)	C32—C33—C34	119.9 (4)
O10^ii^—Cd2—C16^ii^	27.42 (9)	C32—C33—H33A	120.1
O1—C1—O2	124.3 (3)	C34—C33—H33A	120.1
O1—C1—C2	120.1 (3)	C33—C34—C44	117.0 (4)
O2—C1—C2	115.6 (3)	C33—C34—C35	123.8 (4)
C7—C2—C3	120.5 (3)	C44—C34—C35	119.1 (4)
C7—C2—C1	119.4 (3)	N7—C35—C38	120.8 (5)
C3—C2—C1	120.0 (3)	N7—C35—C34	118.1 (6)
C2—C3—C4	119.2 (3)	C38—C35—C34	121.1 (4)
C2—C3—H3A	120.4	N7—C36—C37	123.5 (6)
C4—C3—H3A	120.4	N7—C36—H36A	118.3
C5—C4—C3	120.1 (3)	C37—C36—H36A	118.3
C5—C4—C8	119.4 (3)	N8—C37—C36	121.6 (6)
C3—C4—C8	120.5 (3)	N8—C37—H37A	119.2
C6—C5—C4	120.3 (3)	C36—C37—H37A	119.2
C6—C5—H5A	119.9	N8—C38—C35	122.7 (5)
C4—C5—H5A	119.9	N8—C38—C39	117.3 (5)
O3—C6—C5	118.4 (3)	C35—C38—C39	120.0 (4)
O3—C6—C7	121.6 (3)	C40—C39—C43	117.4 (4)
C5—C6—C7	120.0 (3)	C40—C39—C38	123.2 (4)
C6—C7—C2	119.9 (3)	C43—C39—C38	119.3 (4)
C6—C7—H7A	120.0	C41—C40—C39	119.7 (4)
C2—C7—H7A	120.0	C41—C40—H40A	120.2
O5—C8—O4	121.7 (3)	C39—C40—H40A	120.2
O5—C8—C4	119.4 (3)	C40—C41—C42	119.5 (4)
O4—C8—C4	118.9 (3)	C40—C41—H41A	120.2
O5—C8—Cd1^i^	61.56 (18)	C42—C41—H41A	120.2
O4—C8—Cd1^i^	60.48 (17)	N6—C42—C41	121.9 (4)
C4—C8—Cd1^i^	174.5 (3)	N6—C42—H42A	119.1
O6—C9—O7	122.8 (3)	C41—C42—H42A	119.1
O6—C9—C10	120.6 (3)	N6—C43—C39	122.0 (3)
O7—C9—C10	116.6 (3)	N6—C43—C44	117.4 (3)
C11—C10—C15	120.3 (3)	C39—C43—C44	120.6 (3)
C11—C10—C9	118.7 (3)	N5—C44—C34	122.4 (4)
C15—C10—C9	120.8 (3)	N5—C44—C43	117.7 (3)
C12—C11—C10	120.0 (3)	C34—C44—C43	119.9 (4)
C12—C11—H11A	120.0	C17—N1—C30	118.9 (3)
C10—C11—H11A	120.0	C17—N1—Cd1	124.1 (2)
O8—C12—C13	118.9 (3)	C30—N1—Cd1	115.3 (2)
O8—C12—C11	121.4 (3)	C28—N2—C29	119.1 (3)
C13—C12—C11	119.7 (3)	C28—N2—Cd1	123.2 (3)
C12—C13—C14	120.2 (3)	C29—N2—Cd1	116.6 (2)
C12—C13—H13A	119.9	C22—N3—C21	115.1 (6)
C14—C13—H13A	119.9	C23—N4—C24	115.0 (6)
C15—C14—C13	120.6 (3)	C31—N5—C44	119.5 (3)
C15—C14—C16	120.3 (3)	C31—N5—Cd2	123.0 (3)
C13—C14—C16	119.1 (3)	C44—N5—Cd2	117.4 (2)
C14—C15—C10	119.1 (3)	C42—N6—C43	119.3 (3)
C14—C15—H15A	120.4	C42—N6—Cd2	124.0 (2)
C10—C15—H15A	120.4	C43—N6—Cd2	116.7 (2)
O10—C16—O9	121.5 (3)	C36—N7—C35	115.4 (7)
O10—C16—C14	119.6 (3)	C37—N8—C38	116.0 (7)
O9—C16—C14	118.9 (3)	C1—O1—Cd1	139.9 (2)
O10—C16—Cd2^ii^	61.58 (18)	C1—O2—Cd2	116.7 (2)
O9—C16—Cd2^ii^	60.23 (17)	C6—O3—H3B	109.5
C14—C16—Cd2^ii^	175.5 (2)	C8—O4—Cd1^i^	91.74 (19)
N1—C17—C18	122.7 (4)	C8—O5—Cd1^i^	91.1 (2)
N1—C17—H17A	118.6	C9—O6—Cd2	142.2 (2)
C18—C17—H17A	118.6	C9—O7—Cd1	112.1 (2)
C19—C18—C17	118.8 (4)	C12—O8—H8A	109.5
C19—C18—H18A	120.6	C16—O9—Cd2^ii^	91.65 (19)
C17—C18—H18A	120.6	C16—O10—Cd2^ii^	91.0 (2)
C18—C19—C20	119.7 (4)	H1WA—O1W—H1WB	120.0
C18—C19—H19A	120.1	H2WA—O2W—H2WB	120.0
			
O1—C1—C2—C7	154.7 (3)	O1—Cd1—N1—C17	−107.3 (3)
O2—C1—C2—C7	−26.3 (5)	N2—Cd1—N1—C17	179.4 (3)
O1—C1—C2—C3	−29.3 (5)	O4^i^—Cd1—N1—C17	97.0 (3)
O2—C1—C2—C3	149.8 (3)	O5^i^—Cd1—N1—C17	86.3 (3)
C7—C2—C3—C4	2.3 (5)	C8^i^—Cd1—N1—C17	89.0 (3)
C1—C2—C3—C4	−173.8 (3)	O7—Cd1—N1—C30	−169.8 (2)
C2—C3—C4—C5	−2.6 (5)	O1—Cd1—N1—C30	87.8 (3)
C2—C3—C4—C8	177.6 (3)	N2—Cd1—N1—C30	14.5 (2)
C3—C4—C5—C6	1.4 (5)	O4^i^—Cd1—N1—C30	−67.9 (3)
C8—C4—C5—C6	−178.7 (3)	O5^i^—Cd1—N1—C30	−78.5 (2)
C4—C5—C6—O3	179.7 (3)	C8^i^—Cd1—N1—C30	−75.8 (2)
C4—C5—C6—C7	0.1 (6)	C27—C28—N2—C29	0.5 (7)
O3—C6—C7—C2	179.9 (3)	C27—C28—N2—Cd1	168.1 (4)
C5—C6—C7—C2	−0.5 (6)	C25—C29—N2—C28	1.6 (5)
C3—C2—C7—C6	−0.7 (5)	C30—C29—N2—C28	−178.9 (3)
C1—C2—C7—C6	175.3 (3)	C25—C29—N2—Cd1	−166.8 (3)
C5—C4—C8—O5	−17.3 (5)	C30—C29—N2—Cd1	12.7 (4)
C3—C4—C8—O5	162.5 (3)	O7—Cd1—N2—C28	150.5 (6)
C5—C4—C8—O4	162.5 (3)	O1—Cd1—N2—C28	47.7 (3)
C3—C4—C8—O4	−17.7 (5)	O4^i^—Cd1—N2—C28	−46.6 (3)
C5—C4—C8—Cd1^i^	−116 (2)	N1—Cd1—N2—C28	177.9 (3)
C3—C4—C8—Cd1^i^	64 (3)	O5^i^—Cd1—N2—C28	−101.1 (3)
O6—C9—C10—C11	156.7 (3)	C8^i^—Cd1—N2—C28	−74.5 (3)
O7—C9—C10—C11	−24.2 (5)	O7—Cd1—N2—C29	−41.6 (8)
O6—C9—C10—C15	−27.4 (5)	O1—Cd1—N2—C29	−144.5 (3)
O7—C9—C10—C15	151.7 (3)	O4^i^—Cd1—N2—C29	121.3 (2)
C15—C10—C11—C12	−0.3 (5)	N1—Cd1—N2—C29	−14.3 (2)
C9—C10—C11—C12	175.6 (3)	O5^i^—Cd1—N2—C29	66.7 (2)
C10—C11—C12—O8	−178.8 (3)	C8^i^—Cd1—N2—C29	93.3 (3)
C10—C11—C12—C13	−1.2 (6)	C23—C22—N3—C21	0.8 (9)
O8—C12—C13—C14	178.6 (3)	C24—C21—N3—C22	−1.6 (7)
C11—C12—C13—C14	1.0 (6)	C20—C21—N3—C22	179.9 (5)
C12—C13—C14—C15	0.7 (5)	C22—C23—N4—C24	0.4 (10)
C12—C13—C14—C16	180.0 (3)	C21—C24—N4—C23	−1.2 (8)
C13—C14—C15—C10	−2.3 (5)	C25—C24—N4—C23	178.2 (5)
C16—C14—C15—C10	178.5 (3)	C32—C31—N5—C44	−0.3 (6)
C11—C10—C15—C14	2.0 (5)	C32—C31—N5—Cd2	−176.9 (4)
C9—C10—C15—C14	−173.8 (3)	C34—C44—N5—C31	−0.7 (5)
C15—C14—C16—O10	162.0 (3)	C43—C44—N5—C31	179.3 (3)
C13—C14—C16—O10	−17.3 (5)	C34—C44—N5—Cd2	176.1 (3)
C15—C14—C16—O9	−19.1 (5)	C43—C44—N5—Cd2	−4.0 (4)
C13—C14—C16—O9	161.7 (3)	O2—Cd2—N5—C31	123.5 (4)
C15—C14—C16—Cd2^ii^	58 (3)	O6—Cd2—N5—C31	59.0 (3)
C13—C14—C16—Cd2^ii^	−121 (3)	O9^ii^—Cd2—N5—C31	−37.7 (3)
N1—C17—C18—C19	0.7 (7)	N6—Cd2—N5—C31	−179.7 (3)
C17—C18—C19—C20	0.7 (7)	O10^ii^—Cd2—N5—C31	−94.2 (3)
C18—C19—C20—C30	−1.1 (7)	C16^ii^—Cd2—N5—C31	−66.7 (3)
C18—C19—C20—C21	178.4 (4)	O2—Cd2—N5—C44	−53.1 (4)
C30—C20—C21—N3	176.8 (4)	O6—Cd2—N5—C44	−117.7 (3)
C19—C20—C21—N3	−2.7 (6)	O9^ii^—Cd2—N5—C44	145.7 (2)
C30—C20—C21—C24	−1.8 (6)	N6—Cd2—N5—C44	3.6 (2)
C19—C20—C21—C24	178.8 (4)	O10^ii^—Cd2—N5—C44	89.2 (2)
N3—C22—C23—N4	−0.2 (12)	C16^ii^—Cd2—N5—C44	116.7 (2)
N3—C21—C24—N4	1.9 (7)	C41—C42—N6—C43	0.3 (6)
C20—C21—C24—N4	−179.6 (4)	C41—C42—N6—Cd2	−179.8 (3)
N3—C21—C24—C25	−177.5 (4)	C39—C43—N6—C42	1.8 (5)
C20—C21—C24—C25	1.0 (6)	C44—C43—N6—C42	−178.1 (3)
N4—C24—C25—C29	−179.1 (4)	C39—C43—N6—Cd2	−178.0 (3)
C21—C24—C25—C29	0.3 (6)	C44—C43—N6—Cd2	2.0 (4)
N4—C24—C25—C26	1.8 (7)	O2—Cd2—N6—C42	−19.2 (3)
C21—C24—C25—C26	−178.8 (5)	O6—Cd2—N6—C42	−112.5 (3)
C29—C25—C26—C27	0.6 (8)	N5—Cd2—N6—C42	177.2 (3)
C24—C25—C26—C27	179.7 (5)	O9^ii^—Cd2—N6—C42	89.6 (3)
C25—C26—C27—C28	1.3 (9)	O10^ii^—Cd2—N6—C42	77.1 (3)
C26—C27—C28—N2	−1.9 (9)	C16^ii^—Cd2—N6—C42	80.0 (3)
C26—C25—C29—N2	−2.1 (6)	O2—Cd2—N6—C43	160.6 (2)
C24—C25—C29—N2	178.8 (4)	O6—Cd2—N6—C43	67.4 (3)
C26—C25—C29—C30	178.3 (4)	N5—Cd2—N6—C43	−2.9 (2)
C24—C25—C29—C30	−0.8 (5)	O9^ii^—Cd2—N6—C43	−90.5 (3)
C19—C20—C30—N1	0.3 (6)	O10^ii^—Cd2—N6—C43	−103.0 (2)
C21—C20—C30—N1	−179.2 (3)	C16^ii^—Cd2—N6—C43	−100.1 (2)
C19—C20—C30—C29	−179.2 (4)	C37—C36—N7—C35	0.8 (10)
C21—C20—C30—C29	1.2 (5)	C38—C35—N7—C36	−1.9 (7)
N2—C29—C30—N1	0.9 (5)	C34—C35—N7—C36	178.5 (5)
C25—C29—C30—N1	−179.5 (3)	C36—C37—N8—C38	−1.4 (10)
N2—C29—C30—C20	−179.6 (3)	C35—C38—N8—C37	0.3 (8)
C25—C29—C30—C20	0.0 (5)	C39—C38—N8—C37	−179.4 (5)
N5—C31—C32—C33	1.1 (8)	O2—C1—O1—Cd1	−83.6 (5)
C31—C32—C33—C34	−0.9 (8)	C2—C1—O1—Cd1	95.4 (4)
C32—C33—C34—C44	−0.1 (7)	O7—Cd1—O1—C1	−1.5 (4)
C32—C33—C34—C35	−179.0 (5)	N2—Cd1—O1—C1	169.4 (4)
C33—C34—C35—N7	−2.4 (7)	O4^i^—Cd1—O1—C1	−91.7 (4)
C44—C34—C35—N7	178.7 (4)	N1—Cd1—O1—C1	105.0 (4)
C33—C34—C35—C38	177.9 (5)	O5^i^—Cd1—O1—C1	−101.7 (4)
C44—C34—C35—C38	−1.0 (6)	C8^i^—Cd1—O1—C1	−93.3 (4)
N7—C36—C37—N8	0.9 (12)	O1—C1—O2—Cd2	7.4 (5)
N7—C35—C38—N8	1.4 (7)	C2—C1—O2—Cd2	−171.6 (2)
C34—C35—C38—N8	−178.9 (4)	O6—Cd2—O2—C1	61.2 (3)
N7—C35—C38—C39	−178.9 (4)	N5—Cd2—O2—C1	−2.2 (5)
C34—C35—C38—C39	0.8 (6)	O9^ii^—Cd2—O2—C1	159.5 (3)
N8—C38—C39—C40	1.0 (6)	N6—Cd2—O2—C1	−55.1 (3)
C35—C38—C39—C40	−178.8 (4)	O10^ii^—Cd2—O2—C1	−144.8 (3)
N8—C38—C39—C43	−179.8 (4)	C16^ii^—Cd2—O2—C1	−172.3 (3)
C35—C38—C39—C43	0.4 (6)	O5—C8—O4—Cd1^i^	−6.4 (4)
C43—C39—C40—C41	0.3 (7)	C4—C8—O4—Cd1^i^	173.7 (3)
C38—C39—C40—C41	179.5 (4)	O4—C8—O5—Cd1^i^	6.4 (4)
C39—C40—C41—C42	1.7 (7)	C4—C8—O5—Cd1^i^	−173.8 (3)
C40—C41—C42—N6	−2.1 (7)	O7—C9—O6—Cd2	−93.2 (5)
C40—C39—C43—N6	−2.1 (5)	C10—C9—O6—Cd2	85.9 (5)
C38—C39—C43—N6	178.6 (3)	O2—Cd2—O6—C9	10.2 (4)
C40—C39—C43—C44	177.8 (4)	N5—Cd2—O6—C9	172.6 (4)
C38—C39—C43—C44	−1.5 (5)	O9^ii^—Cd2—O6—C9	−84.4 (4)
C33—C34—C44—N5	0.9 (6)	N6—Cd2—O6—C9	108.8 (4)
C35—C34—C44—N5	179.9 (3)	O10^ii^—Cd2—O6—C9	−93.1 (5)
C33—C34—C44—C43	−179.0 (4)	C16^ii^—Cd2—O6—C9	−85.2 (4)
C35—C34—C44—C43	−0.1 (5)	O6—C9—O7—Cd1	7.5 (4)
N6—C43—C44—N5	1.3 (5)	C10—C9—O7—Cd1	−171.6 (2)
C39—C43—C44—N5	−178.6 (3)	O1—Cd1—O7—C9	64.0 (3)
N6—C43—C44—C34	−178.8 (3)	N2—Cd1—O7—C9	−38.6 (8)
C39—C43—C44—C34	1.3 (5)	O4^i^—Cd1—O7—C9	158.3 (3)
C18—C17—N1—C30	−1.5 (6)	N1—Cd1—O7—C9	−64.8 (3)
C18—C17—N1—Cd1	−165.9 (3)	O5^i^—Cd1—O7—C9	−146.8 (3)
C20—C30—N1—C17	1.0 (5)	C8^i^—Cd1—O7—C9	−174.0 (3)
C29—C30—N1—C17	−179.4 (3)	O10—C16—O9—Cd2^ii^	−6.2 (4)
C20—C30—N1—Cd1	166.7 (3)	C14—C16—O9—Cd2^ii^	174.9 (3)
C29—C30—N1—Cd1	−13.8 (4)	O9—C16—O10—Cd2^ii^	6.1 (4)
O7—Cd1—N1—C17	−5.0 (3)	C14—C16—O10—Cd2^ii^	−175.0 (3)

Symmetry codes: (i) −*x*, −*y*, −*z*; (ii) −*x*+1, −*y*, −*z*+1.

### Hydrogen-bond geometry (Å, º)

**Table:**

*D*—H···*A*	*D*—H	H···*A*	*D*···*A*	*D*—H···*A*
O3—H3*B*···O2*W*^iii^	0.82	1.84	2.659 (4)	177
O8—H8*A*···O1*W*^iv^	0.82	1.85	2.672 (4)	177
O1*W*—H1*WA*···O9^v^	0.85	2.14	2.912 (4)	150
O1*W*—H1*WB*···O2^v^	0.85	2.27	2.963 (4)	138
O2*W*—H2*WA*···O4^vi^	0.85	2.28	2.880 (4)	127
O2*W*—H2*WB*···O7^vi^	0.85	2.33	2.955 (4)	131

Symmetry codes: (iii) *x*, *y*−1, *z*; (iv) *x*−1, *y*−1, *z*; (v) *x*, *y*+1, *z*; (vi) *x*+1, *y*+1, *z*.
